# Anti-biofilm activity of *Pseudomonas fluorescens* culture supernatants on biofilm formation of *Staphylococcus epidermidis* 1457

**DOI:** 10.1186/s13104-022-06257-z

**Published:** 2022-12-12

**Authors:** Euna Choi, Bethany Wells, Gabrielle Mirabella, Emilee Atkins, Sunga Choi

**Affiliations:** 1 Biology Department, Union University, 1050 Union University Drive, Jackson, TN 38305 USA; 2 Department of Bioinformatics and Biosystems, Seongnam-Campus of Korea Polytechnics, Seongnam, South Korea

**Keywords:** *Staphylococcus epidermidis*, Biofilm, *Pseudomonas fluorescens*, Culture supernatant, Polysaccharide intercellular adhesin

## Abstract

**Objective:**

*Staphylococcus epidermidis* is a skin colonizer and a major cause of nosocomial infections that can lead to sepsis. It causes opportunistic infections by forming biofilms on medical devices, which are hard to control with conventional antibiotics. In an attempt to develop its biofilm inhibitors, the culture supernatant (CS) of *Pseudomonas fluorescens* was assessed. This study examined the effect of the CS on *S. epidermidis* 1457 biofilm formation, the characteristics of inhibitors in the CS, and the differential gene expression of *S. epidermidis* when treated with the CS.

**Results:**

*P. fluorescens* CS specifically targeted the maturation stage of *S. epidermidis* biofilm formation while not affecting planktonic growth. RT-qPCR analysis revealed that *P. fluorescens* CS significantly downregulated *S. epidermidis ica* genes and upregulated an *ica* repressor, *tcaR*. This indicates that the CS reduced polysaccharide intercellular adhesin synthesis, a major component of the *S. epidermidis* 1457 biofilm matrix. Further studies are required to elucidate the specific inhibitory components in the CS and their mechanism of action. Our results indicate that inhibitors in the *P. fluorescens* CS may have a significant value for inhibiting *S. epidermidis* biofilm. Combinations of specific inhibitors from the CS and antibiotics against staphylococci may provide an effective measure to control *S. epidermidis* biofilm formation while avoiding antibiotic resistance and compensating the attenuated effectiveness of antibiotics on biofilms.

**Supplementary Information:**

The online version contains supplementary material available at 10.1186/s13104-022-06257-z.

## Introduction

*Staphylococcus epidermidis* is a commensal bacterium found on human skin and mucous membranes. Along with *Staphylococcus aureus*, *S. epidermidis*-mediated infections are becoming increasingly prevalent as indwelling medical devices are more commonly used. They are often associated with hip implants and catheters, causing systemic infections [1].

*S. epidermidis* does not express as many virulence factors as *S. aureus* [2]. One important molecular mechanism that triggers the transition of commensal *S. epidermidis* to an opportunistic pathogen is the ability to form biofilms [3, 4]. Polysaccharide intercellular adhesin (PIA) composed of ß-1,6-*N*-acetyl-_D_-glucosamine polymers (PNAG) is a major component of *S. epidermidis* biofilm. PIA, proteins, and extracellular DNA are adhesion molecules and make up the extracellular polymeric substances (EPS) in the biofilm matrix [5]. With enhanced PIA production, the established biofilms clog medical devices, attenuating antibiotic penetration and host immune response [3, 6].

Taking into consideration the increasing prevalence of biofilm-mediated infections, it is critical to understand the mechanisms of *S. epidermidis* biofilm formation and develop methods of inhibition. Previously, extracellular products of *Pseudomonas aeruginosa* and the CS of some *Actinomycete* and *Enterobacter* strains were demonstrated to inhibit *S. epidermidis* biofilm formation [7–9].

*P. fluorescens,* soil-associated bacteria, suppress plant diseases by producing antimicrobial substances [10]. Recently, the quorum sensing (QS) molecules of *P. fluorescens* has been shown to inhibit biofilm formation of *Shewanella baltica* [11]. In this study, the effects of *P. fluorescens* CS were assessed to determine whether *P. fluorescens* produce antibiofilm agents against *S. epidermidis* in addition to previously known bioactive agents. In vitro biofilm assays and RT-qPCR revealed that *P. fluorescens* CS functions against *S. epidermidis* biofilm formation and influences genes involved in PIA production, indicating the presence of anti-biofilm agents in the CS.

## Methods

### Bacterial strains and preparation of bacterial CS for biofilm assay

*S. epidermidis* 1457, kindly donated by Dr. Paul Fey (University of Nebraska Medical Center), was cultured in tryptic soy broth (TSB) at 30 °C. *P. fluorescens* Pf01 strain was a gift from Dr. George O’Toole (Dartmouth University) and was maintained in Luria-Bertani broth (LB) at 30 °C. To prepare the CS, an overnight *P. fluorescens* culture was used to inoculate 200 ml LB (starting OD_660_ = 0.05). The culture was grown at 30 °C with agitation to the early-stationary phase (OD_660_ = 0.8), centrifuged at 4 °C, 12,000 g for 15 min, passed through a 0.22 µm filter system, and then heat-treated (100 °C) for 5 min for all biofilm assays.

### Characterization of inhibitory factors in *P. fluorescens* CS

The filtered CS was subject to one of the following. 1. Heating at 100 °C for 1–5 min. 2. Proteinase K (400 µg/mL) treatment at 55 °C, 30 min. 3. Trypsin (2.5 µg/mL) treatment at 37 °C for 4 h [12]. 4. Cellulase (5 mg/ml, MP Biomedicals) treatment at 37 °C for 1 h [7]. 5. Lipase acrylic resin (2 mg/mL, Millipore Sigma, L4777) treatment at 37 °C for 48 h [13]. 6. Heating at 100 °C for 5 min and centrifuging in a Centricon tube (Millipore) with a 3 K molecular weight (MW) limit (7500X g, 40 min at 4 °C, [13]). Enzyme treatments of CS were performed with gentle shaking, followed by heat inactivation at 95 °C for 20 min.

### Growth kinetics and biofilm assay of *S. epidermidis*

Overnight cultures of *S. epidermidis* were prepared to reach the exponential growth phase (OD_600_ = 0.8, ≈1X 10^9^ CFU/mL), and diluted with TSB (1:5000). The diluted cultures (200 µl) were incubated with or without 50% (v/v) *P. fluorescens* CS in a 48-well plate (Costar, polystyrene) and monitored using a microtiter plate reader (BioTek) for 72 h to analyze growth kinetics. For the biofilm assays, after 24 h incubation without agitation, the plate was washed, stained with 0.1% crystal violet, and read at optical density 600 nm [7].

### Biofilm disruption assay

Biofilm disruption was performed with NaIO_4_, Proteinase K, or DNase I. *S. epidermidis* was allowed to form biofilm with or without 25% (v/v) *P. fluorescens* CS at 30 °C for 16 h. After washing, the preformed biofilm was treated with either NaIO_4_ (40 mM in dH2O) for 2 h at 37 °C, Proteinase K (0.1 mg/mL, in 20 mM Tris-HCl, 1 mM CaCl_2_, pH 7.5), or DNase I (0.5 mg/mL in 5 mM MgCl_2_, DNA 25, Sigma) for 24 h at 37 °C [14–16]. After incubation with each disruptive agent, the remaining biofilms were quantified as above after crystal violet staining.

### Reverse transcription-quantitative PCR

*S. epidermidis* biofilm was lysed with a sonicator (Fisher Scientific) at amplitude 4.0 for 20 s and treated with lysozyme (50 mg/mL, 2 h, 37 °C). Total RNA was isolated using the RiboPure Bacteria and treated with DNase I (Invitrogen). cDNA was generated from total RNA (1 µg) using the SuperScript III first-strand synthesis system (Invitrogen). qPCR was performed using cDNA (diluted 1:20), Advanced Universal SYBR green Supermix (Bio-Rad), Bio-Rad CFX96 Touch real-time PCR detection systems, and primers (Additional file 1: Table S1). All data were analyzed with Bio-Rad CFX manager software 3.1 using Cq values. 16S rRNA was used for normalization. The mean fold change between study groups was calculated following the 2^−ΔΔCt^ method [17].

### Statistical analysis

Three independent experiments were performed using 3 biological samples in triplicate. One representative data (n = 9) were analyzed by unpaired T-test or analysis of variance (ANOVA) using Graph Pad Prism 9 (*P*-value < 0.05 is considered statistically significant).

## Results

### *P. fluorescens *CS inhibits *S. epidermidis* biofilm maturation but not its growth

**Fig. 1:**
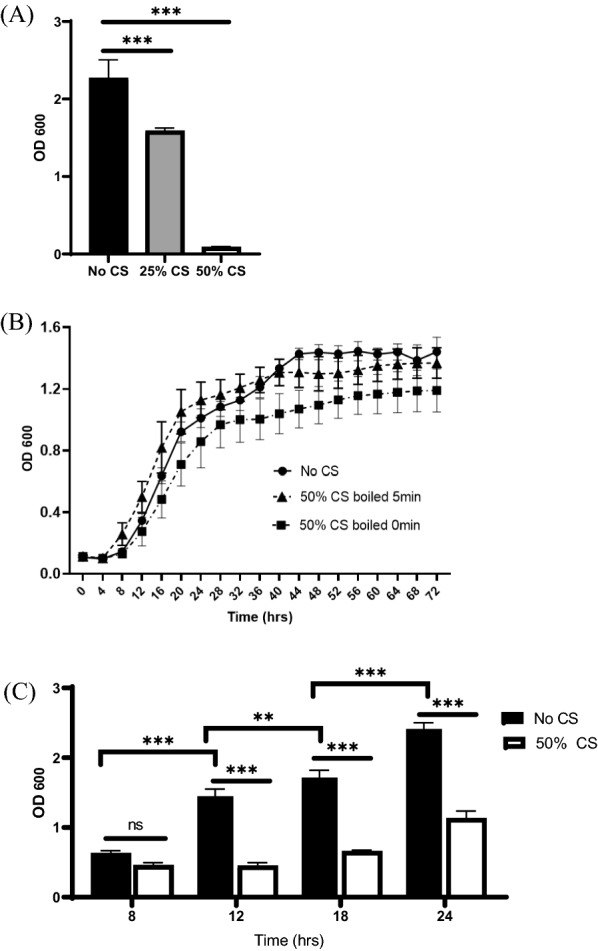
*P. fluorescens* CS specifically inhibits *S. epidermidis* biofilm maturation but not its growth. **A** *S. epidermidis* biofilm assay was performed with 0%, 25%, or 50% addition of *P. fluorescens* CS. **B** The growth kinetics of *S. epidermidis* were measured with or without 50% *P. fluorescens* CS. One representative result from three biological samples is shown as means with standard deviations (n = 3). **C** *S. epidermidis* biofilm with 50% *P. fluorescens* CS were removed at time intervals of 8, 12, 18, or 24 h. The bars indicate standard deviation (n = 9). **, *** indicate statistical significance at P < 0.01 and P < 0.001, respectively

Various concentrations of the *P. fluorescens* CS were tested on *S. epidermidis* biofilm. Addition of 25% and 50% CS resulted in approximately 30% and 95% biofilm reduction, respectively (Fig. 1A). This result indicates that *P. fluorescens* CS effectively inhibited *S. epidermidis* biofilm formation in a concentration-dependent manner. Growth kinetics revealed that boiled *P. fluorescens* CS did not affect bacterial growth of *S. epidermidis*, although untreated CS resulted in slight but noticeable growth retardation after 40 h (Fig. 1B). These results suggest anti-biofilm activity of *P. fluorescens* CS on *S. epidermidis* independent of growth inhibition. Previous studies indicate there are four stages in forming *S. epidermidis* biofilm, including adherence, accumulation, maturation, and detachment [4]. Treatment of *S. epidermidis* with *P. fluorescens* CS for 8 h showed no significant change in biofilm formation, whereas treatments for 12, 18, and 24 h showed 70%, 63%, and 54% biofilm inhibition, respectively (Fig. 1C). This indicates that the CS specifically attenuates biofilm maturation stages rather than initial attachment (< 8 h).

### The inhibitory compounds of *P. fluorescens* CS are heat-resistant and multifactorial

**Fig. 2:**
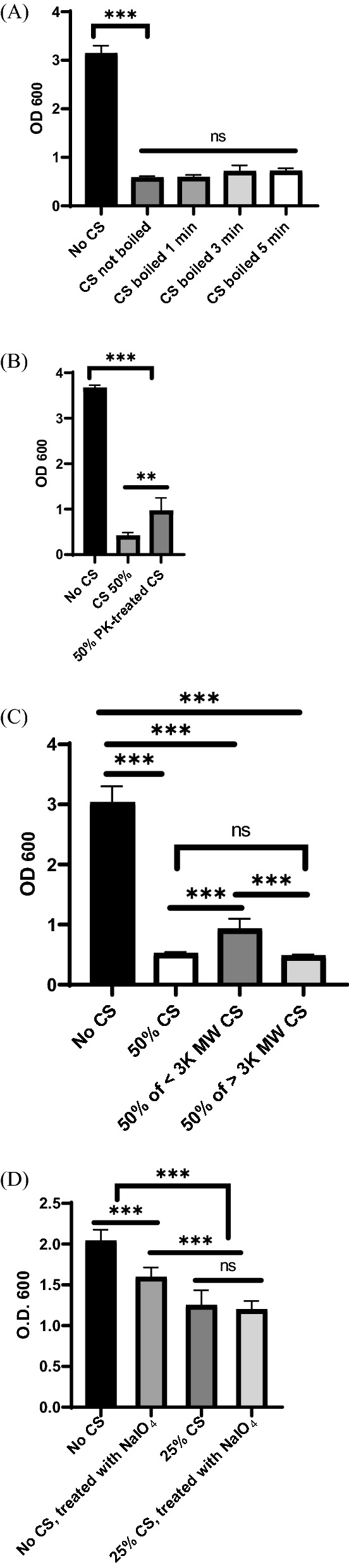
The inhibitory components of *P. fluorescens* CS are heat-resistant and not small molecules. **A** *P. fluorescens* CS was boiled for various times up to 5 min before treating *S. epidermidis*. **B** *P. fluorescens* CS was treated with proteinase K and used for *S. epidermidis* biofilm. **C** Boiled *P. fluorescens* CS was filtered in a Millipore Centricon tube with 3 K MW limit (> 3 K MW: retentate; < 3 K MW: filtrate). **D** *S. epidermidis* 1457 biofilm assay was performed with or without 25% CS. The preformed biofilms were treated with NaIO_4_ for disruption. Data shown as means from three separate assays. The bars indicate standard deviation (n = 9). **, *** indicate statistical significance at P < 0.01 and P < 0.001, respectively, ns: not significant

To further characterize the active compound(s) essential for biofilm inhibition, *P. fluorescens* CS was treated with heat or various enzymes. The 5 min-boiled CS showed comparable inhibition with those heated for less time or not at all (< 5 min) (Fig. 2A). Biofilm inhibition also remained the same after treatment of CS with either trypsin, cellulase, or lipase (Additional file 1: Fig. S1A, B, C). However, Proteinase K treatment attenuated the inhibitory function of the CS, showing 15% less biofilm inhibition than untreated CS (Fig. 2B). These results indicate that the inhibitory components in the CS are heat-resistant, and some are proteinaceous. The CS fraction  > 3 k MW showed similar inhibition to whole CS, although the fraction < 3 k MW also inhibited biofilm but at a significantly lower rate than components > 3 k (Fig. 2C). This indicates that there are inhibitory components in the CS that are both larger and smaller than 3 k MW.

### *P. fluorescens* CS targets polysaccharide intercellular adhesin

Preformed biofilms (16 h) were treated with biofilm-degrading agents such as NaIO_4,_ Proteinase K, or DNase I to investigate which component of *S. epidermidis* biofilm was specifically influenced by *P. fluorescens* CS [14–16]. It is reasonable to assume that if a particular macromolecule of the biofilm matrix was targeted by the CS, there would be none left to be further disrupted by a chemical treatment. NaIO_4_ is an oxidizing agent that targets glucose-containing polysaccharides in biofilm [14]. The mature *S. epidermidis* biofilm without the CS was treated with NaIO_4_ and showed approximately 20% biofilm disruption (Fig. 2D). Mature biofilm formed in the presence of 25% CS was not significantly affected by NaIO_4_, implying inhibitors in the *P. fluorescens* CS already removed most of the polysaccharides in the biofilm matrix*.* Similar experiments using Proteinase K showed no noticeable disruption of *S. epidermidis* biofilm, suggesting that proteins are not major components of *S. epidermidis* 1457 biofilm, as previously reported (Additional file 1: Fig. S2A, [18]). The preformed biofilms with or without CS were further disrupted by DNase I, indicating *P. fluorescens* CS did not target nucleic acids in biofilms (Additional file 1: Fig. S2B).

### *P. fluorescens *CS downregulates *S. epidermidis* PIA biosynthesis

**Fig. 3:**
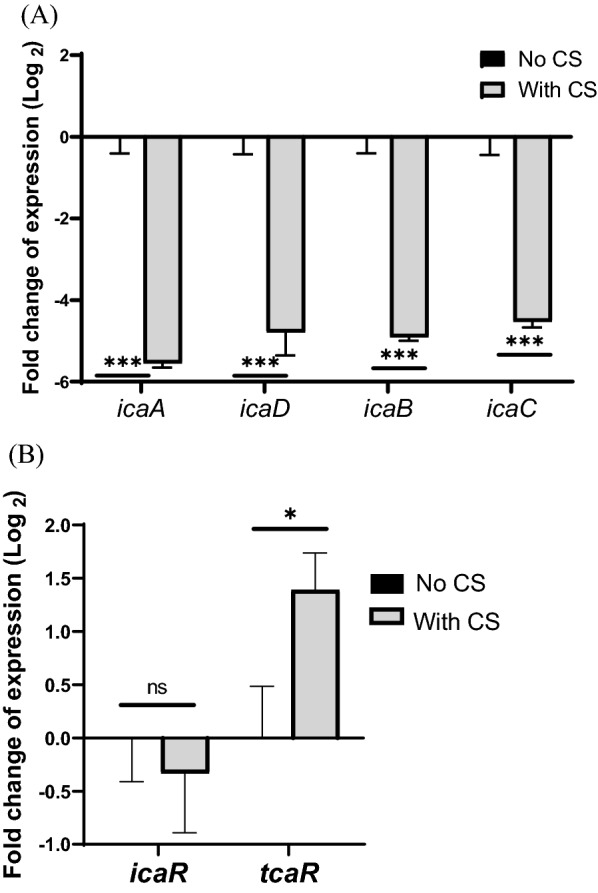
*P. fluorescens* CS differentially regulates genes involved in *S. epidermidis* polysaccharide biosynthesis. **A** RT-qPCR was performed on *icaADBC* from *S. epidermidis* with or without CS. **B** RT-qPCR was performed on *ica* repressors from *S. epidermidis* with or without CS. *, *** indicate statistical significance at P < 0.05 and P < 0.001, respectively

RT-qPCR analysis revealed that CS treatment significantly downregulated the *icaADBC* operon, which is essential for PIA synthesis, and upregulated *tcaR*, a main *ica* repressor in *S. epidermidis* 1457 (Fig. 3A, B, [19]). No other biofilm-related regulatory genes tested showed different expression with CS treatment (Additional file 1: Fig. S3, [15, 20–24]). These results indicate that one of the main inhibitory mechanisms of *P. fluorescens* CS is to regulate *icaADBC* gene expression at the transcriptional level, resulting in significant reduction in PIA, a major component of the *S. epidermidis* 1457 biofilm matrix [18]. Biofilm assay was performed with additional glucose since PIA production and *ica* gene expression have been shown to be affected by glucose addition (Additional file 1: Fig. S4, [25]). Furthermore, *S. epidermidis* biofilm with CS treatment in our biofilm assay has fewer nutrients available than *S. epidermidis* without the CS. Supplementation with different concentrations of glucose did not affect the CS inhibition level, indicating that glucose did not modulate the *ica* operon in our system and the biofilm inhibition observed is not due to a lack of energy sources for PIA synthesis or planktonic growth of *S. epidermidis* with the spent media of *P. fluorescens*.

## Discussion

Biofilm formation is a main mode of *S. epidermidis* pathogenicity which causes opportunistic infections in hospitalized patients, imposing heavy physical and economic burdens. The prevalence and structure of *S. epidermidis* biofilms vary greatly depending on environmental factors such as nutrients, pH, temperature, and multispecies presence, and the seed time of *S. epidermidis* on medical devices [26]. In our study, the CS of *P. fluorescens* strain Pf01 showed specific inhibition of the maturation stages of *S. epidermidis* biofilm formation while not affecting planktonic growth*.* This is partly due to gene regulation at the transcriptional level, affecting the *ica* operon and *tcaR*, resulting in significant reduction in PIA/PNAG synthesis and almost complete biofilm inhibition. Pf01 CS was shown to inhibit *Shewanella baltica* by producing various QS molecules such as acyl-l-homoserine lactones (AHLs) and the autoinducer-2 (AI-2) [9]. Although our liquid chromatography-mass spectrometry (LC-MS) analysis did not reveal the presence of known QS molecules in the CS (data not shown), it is possible that Pf01 CS contains specific QS inhibitors (QSI) against *S. epidermidis*, affecting the expression levels of stress-response genes, including the *ica* operon. The majority of inhibitory components were heat-stable and enzyme-resistant macromolecules (> 3 K MW), which may work together with small-molecule inhibitors like QSIs, displaying robust biofilm inhibition. Importantly, direct cell lysis is not an inhibitory action mode of the CS, since *S. epidermidis* was viable and showed no growth retardation with the boiled CS. This is different from *P. aeruginosa* exopolysaccharides, which have been shown to inhibit both the planktonic growth and biofilm formation of *S. epidermidis* [7]*.* Targeting only biofilm formation without interfering with bacterial growth is an important property in developing biofilm inhibitors since those interfering with cell growth often induce selective pressure on drug-resistant strains, resulting in survival of highly virulent pathogens [27]. To our knowledge, Pf01 is one of few CSs reported to specifically inhibit staphylococcal biofilms without affecting bacterial growth [8]. Other aspects of the CS’s functions are under investigation including removing existing *S. epidermidis* biofilms, regulating biofilm-related accessory genes at different stages of biofilm formation, and inhibiting the formation of other bacterial biofilms. The CS should also be tested on other *S. epidermidis* strains that produce a protein-dependent biofilm matrix. Taken together, the *P. fluorescens* CS is promising as an alternative and complementary measure to minimize staphylococcal biofilm EPS within medical devices, providing better control against *S. epidermidis-*mediated nosocomial infection.

## Limitations

It is not certain whether *S. epidermidis* biofilm inhibition was due to specific inhibitors or nonspecific degradation by metabolic wastes in the CS. Ethyl acetate extraction and LC-MS analysis of *P. fluorescens* CS did not reveal any known QS molecules in Pf01. Assays using biosensor strains for detecting AHLs and AI-2 in the CS will be necessary. Generating mutant strains of *P. fluorescens* and *S. epidermidis* will be performed to determine the precise inhibitory mechanism of *P. fluorescens* CS on *S. epidermidis* biofilm.

## Supplementary Information

**Additional file 1: Figure S1.** S. epidermidis biofilm with enzyme-treated P. fluorescens CS. (A) P. fluorescens CS was pre-treated with trypsin, (B) cellulase, or (C) lipase and used for S. epidermidis biofilm assay. Data shown as means from 3 separate assays. The bars indicate standard deviation (n=9). *** indicate statistical significance at P<0.001, ns: not significant. **Figure S2.** Effects of Proteinase K and DNase on preformed S. epidermidis biofilm with or without CS. (A) S. epidermidis 1457 biofilm assay was performed with or without 25% CS. The preformed biofilms were treated with Proteinase K or (B) DNase for disruption. *** indicate statistical significance at P<0.001, ns: not significant. **Figure S3.** RT-qPCR was performed on multiple biofilm-associated genes from S. epidermidis with or without CS. ns: not significant. **Figure S4.** P. fluorescens CS was supplemented with different concentrations of glucose and used for biofilm assay. *** indicate statistical significance at P<0.001, ns: not significant. **Table S1.** List of primer sequences used for RT-qPCR.

## Data Availability

The datasets used and/or analyzed during the current study are available from the corresponding author on reasonable request.

## Abbreviations

AHL: Acyl-L-homoserine lactone
ANOVA: Analysis of variance
AI-2: Autoinducer-2
CFU: Colony forming units
CS: Culture supernatant
EPS: Extracellular polymeric substances
LB: Luria-Bertani
LC-MS: Liquid chromatography-mass spectrometry
MW: Molecular weight
PIA: Polysaccharide intercellular adhesin
PNAG: ß-1,6-*N*-acetyl-_D_-glucosamine polymers
*P. fluorescens*: *Pseudomonas fluorescens*
QS: Quorum-sensing
QSI: Quorum-sensing inhibitor
RT-qPCR: Reverse transcription-quantitative polymerase chain reaction
*S. epidermidis*: *Staphylococcus epidermidis*
TSB: Tryptic soy broth
