# Supplementary material for: Anti-biofilm activity of Pseudomonas fluorescens culture supernatants on biofilm formation of Staphylococcus epidermidis 1457
Source: BMC Res Notes. 2022 Dec 12;15:370. doi: 10.1186/s13104-022-06257-z (PMC9743590; doi:10.1186/s13104-022-06257-z)
Supplement: Supplementary file 1 — Additional file 1: Figure S1. S. epidermidis biofilm with enzyme-treated P. fluorescens CS. (A) P. fluorescens CS was pre-treated with trypsin, (B) cellulase, or (C) lipase and used for S. epidermidis biofilm assay. Data shown as means from 3 separate assays. The bars indicate standard deviation (n=9). *** indicate statistical significance at P<0.001, ns: not significant. Figure S2. Effects of Proteinase K and DNase on preformed S. epidermidis biofilm with or without CS. (A) S. epidermidis 1457 biofilm assay was performed with or without 25% CS. The preformed biofilms were treated with Proteinase K or (B) DNase for disruption. *** indicate statistical significance at P<0.001, ns: not significant. Figure S3. RT-qPCR was performed on multiple biofilm-associated genes from S. epidermidis with or without CS. ns: not significant. Figure S4. P. fluorescens CS was supplemented with different concentrations of glucose and used for biofilm assay. *** indicate statistical significance at P<0.001, ns: not significant. Table S1. List of primer sequences used for RT-qPCR. [file 13104_2022_6257_MOESM1_ESM.pdf]

Supplemental figures

(A)

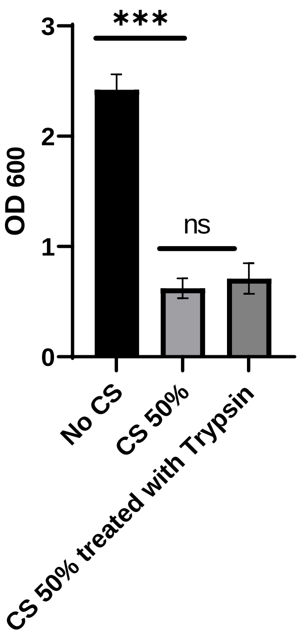

(B)

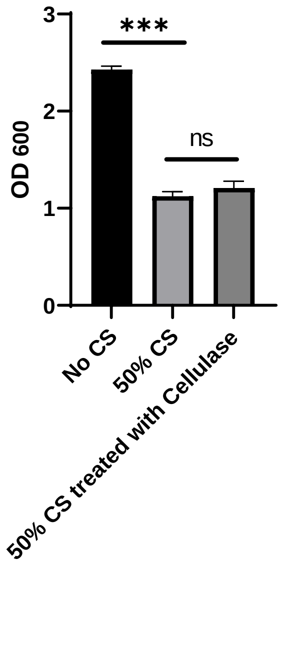

(C)

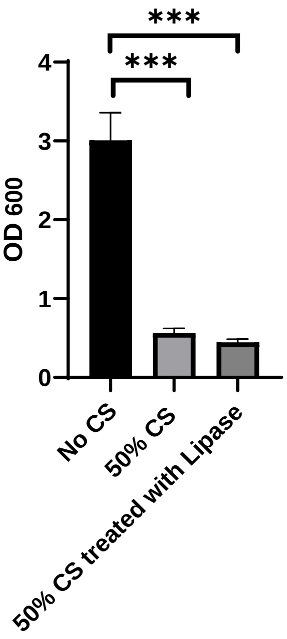

Figure S1. *S. epidermidis* biofilm with enzyme-treated *P. fluorescens* CS.
(A) *P. fluorescens* CS was pre-treated with Trypsin, (B) cellulase, or (C) lipase and used for *S. epidermidis* biofilm. Data shown as means from 3 separate assays. The bars indicate standard deviation (n=9). *** indicate statistical significance at P<0.001, ns: not significant.

(A)

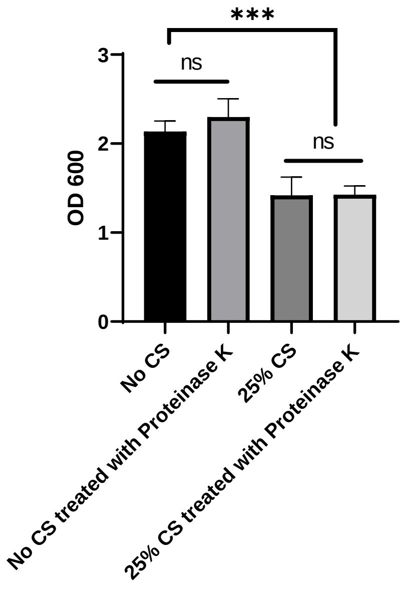

(B)

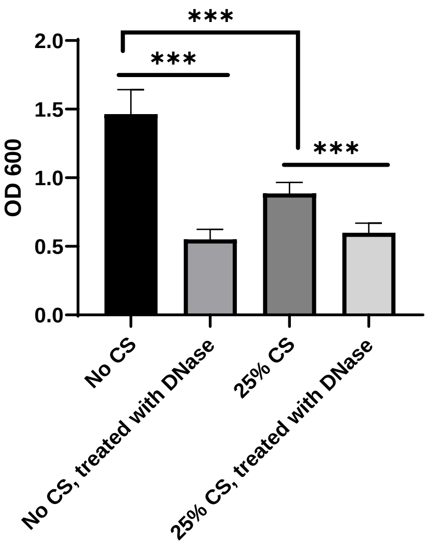

Figure S2. Effects of proteinase K and DNase on preformed *S. epidermidis* biofilm with or without CS.

(A) *S. epidermidis* 1457 biofilm assay was performed with or without 25% CS. The preformed biofilms were treated with Proteinase K or (B) DNase for disruption. *** indicate statistical significance at P<0.001, ns: not significant.

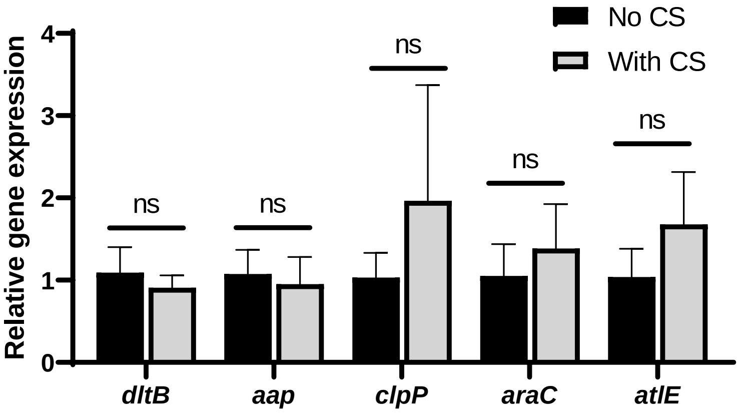

Figure S3. RT-qPCR was performed on multiple biofilm-associated genes from *S. epidermidis* with or without CS. ns: not significant.

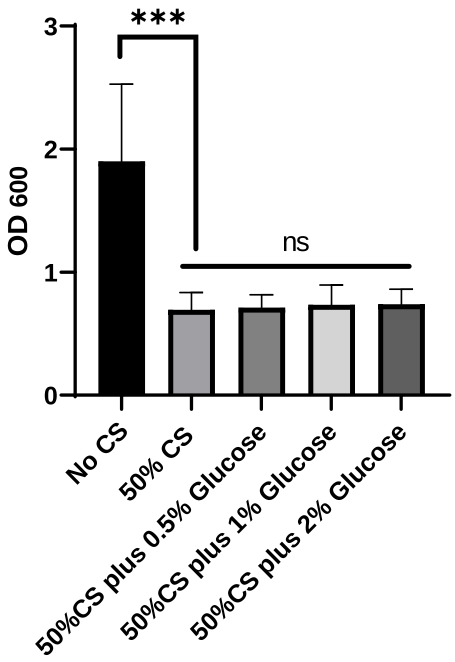

Figure S4. *P. fluorescens* CS was supplemented with different concentration of glucose and used for biofilm assay. *** indicate statistical significance at $P<0.001$, ns: not significant.

Table S1. List of primer sequences used for RT-qPCR.

Gene		Oligonucleotide sequence (5' to 3')
16s rRNA	Fw:	AGGAGTCTGGACCGTGTCTC
	Rv:	GCGTAGCCGACCTGAGAG
icaA	Fw:	TGCACTCAATGAGGGAATCA
	Rv:	TAACTGCGCCTAATTTTGGATT
icaB	Fw:	GAAACAGGCTTATGGGACTTTG
	Rv:	CAAGTGCGCGTTCATTTTT
icaC	Fw:	TTTACGTGCGTTTATTTGTG
	Rv:	GCCGATTAAAAGATATGGAA
icaD	Fw:	ATGGTCAAGCCCAGACAGAG
	Rv:	CAAACAAACTCATCCATCCG
icaR	Fw:	ACTTGGTCTTTAAAACGGTT
	Rv:	CAAACACCATCTTCAAGAAA
tcaR	Fw:	ATGGCATATCAGCTGAGCAA
	Rv:	CAGCATTGAGCAACTTTTTGA
dltB	Fw:	TGGCATGGTCTAGAAGTATATTACATTGT
	Rv:	CCAACGCTCATAATAACCATAACCT
aap	Fw:	GCACCAGCTGTTGTTGTACC
	Rv:	GCATGCCTGCTGATAGTTCA
clp	Fw:	CAAACCAGACGTTCAAACAATCTG
	Rv:	AGAATGAACCCATAGACGCTGCCATACCAATT
araC	Fw:	GGAAACCAGCTATTTGACCGATT
	Rv:	AAAACTCCCCTTACTAAACCACACAT
atlE	Fw:	AAAGAAACGGCATCTAAT
	Rv:	TTCTTCAATTCTTGGTGCTT
